# Deposition of Doxorubicin in Rats following Administration of Three Newly Synthesized Doxorubicin Conjugates

**DOI:** 10.1155/2013/926584

**Published:** 2013-12-05

**Authors:** Menglei Huan, Shuang Tian, Han Cui, Bangle Zhang, Dan Su, Jieping Wang, Kangchu Li, Weidong Cao

**Affiliations:** ^1^Department of Pharmaceutics, School of Pharmacy, Fourth Military Medical University, Xi'an, Shaan'xi 710032, China; ^2^Department of Obstetrics & Gynecology, PLA General Hospital, Beijing 100853, China; ^3^Department of Clinic Pharmacology, Wuhan General Hospital of Guangzhou Military Area Command, Wuhan, Hubei 430070, China; ^4^Department of Pharmacy, Tangdu Hospital, Fourth Military Medical University, Xi'an, Shaan'xi 710038, China; ^5^Department of Radiation Medicine, School of Public Health, Fourth Military Medical University, Xi'an, Shaan'xi 710032, China; ^6^Department of Neurosurgery, Xijing Hospital, Fourth Military Medical University, Xi'an, Shaan'xi 710032, China

## Abstract

We previously reported the synthesis of three DOX conjugates that represented different targeting vehicles and showed them to have antitumor activity both *in vitro* and *in vivo*. However, the relationships between the pharmacokinetics of these DOX conjugates and their chemical structures were not characterized. In the current study, free DOX derived from each of the conjugates was found at low levels in the rat circulatory system, with conjugated DOX being the major form. The two polyethylene glycol (PEG) conjugates slowly released DOX, and *t*_1/2_ **β** for total DOX from DOX-LNA, PEG-ami-DOX, and PEG-hyd-DOX was 5.79, 10.22, and 15.18 h, respectively. All three conjugates also deposited less DOX into normal organs than did an equivalent dose of free DOX, and the C_max_ value of free DOX released by DOX- LNA, PEG-ami-DOX, and PEG-hyd-DOX was 32.5, 9.5, and 4.7 **μ**g/g, respectively. Among the conjugates, the compound with an acid-labile bond between PEG and DOX exhibited the lowest free DOX deposition in healthy tissues, which should decrease the systemic toxicity of free DOX while allowing for tumor targeting by PEG.

## 1. Introduction

Doxorubicin (DOX) is a widely used chemotherapeutic agent. However, owing to its severe toxic side effects, for example, cardiomyopathy, caused mainly by its low selectivity, its clinical use has been limited [1–3]. Given this problem, many tumor-targeting delivery systems have been tested to improve the therapeutic efficacy of DOX and reduce its side effects [4]. Two basic types of tumor-targeting delivery systems—active and passive—have been assessed. High-molecular-weight carriers for example, liposomes, microspheres, and micelles, have been widely used as passive drug-delivery systems owing to their EPR effect [5, 6], whereas anticancer drugs or drug carriers that are conjugated to a tumor-specific targeting moiety [7–9], for example, *α*-linolenic acid and folate, actively target tumors. Although targeting systems for DOX have been developed and at least one is in a clinical trial [10], no formulations have been marketed. Given that the tested DOX tumor-targeting systems have been shown to lose their tumor-targeting ability and consequently promote severe toxicity *in vivo*, but how to reduce this toxicity is a current problem. A DOX delivery system should have a low (or no) distribution in normal tissues and be stable prior to delivery. Therefore, determination of the pharmacokinetics and tissue distribution of DOX before and after release from a carrier needs to be evaluated.

Analytical methods, including HPLC with UV or fluorescence monitoring, have been used to determine free DOX levels in its clinical samples [11, 12]. However, HPLC-MS/MS is more suitable for pharmacokinetic and bio-distribution investigations of DOX owing to the technique's high sensitivity and specificity, which allows for picogram quantification of DOX from biological samples. Furthermore, HPLC/MS-MS offers other advantages than those associated with traditional analytical methods, including small sample volumes and rapid assay times [13, 14].

We previously synthesized three DOX conjugates, namely, DOX-LNA, PEG-ami-DOX, and PEG-hyd-DOX, having different antitumor efficacies [15–17]. However, the relationships between the pharmacokinetics and the chemical structures of these compounds have not been characterized. For this report, we present a reproducible, rapid, sensitive, and specific LC-ESI-MS/MS method for determining free DOX from blood and solid-organ samples. We validated the method using healthy rats that had received an intravenous injection of free or conjugated DOX. We found the three DOX conjugates to have different pharmacokinetics that seem related to their chemical structures. All DOX conjugate-treated rats had less free DOX present in their circulatory systems and organs than did rats treated with free DOX. DOX-LNA showed the largest release of DOX possibly owing to its smaller molecular weight. PEG-hyd-DOX released the least amount of DOX probably because it incorporates an acid-sensitive bond between DOX and PEG. Our results suggest that PEG-hyd-DOX has potential as a chemotherapeutic DOX-delivery system because it has a good antitumor performance and low toxicity.

## 2. Materials and Methods

### 2.1. Chemicals and Reagents

Doxorubicin (>99% purity) was obtained from Sigma-Aldrich (Louis, MO, USA). Resveratrol (>99% purity) was purchased from the National Institute for the Control of Pharmaceutical and Biological Products (Beijing, China). HPLC-grade acetonitrile, methanol, and formic acid were purchased from Tedia Company, Inc (Fairfield, OH, USA). All other reagents and chemicals were analytical grade. Water was purified using a Milli-Q system (Millipore Co., Milford, MA, USA). DOX-LNA, PEG-ami-DOX, and PEG-hyd-DOX were synthesized in our previously experiments.

### 2.2. Animals

The Ethics Committee of the Fourth Military Medical University approved the animal protocol. Sprague-Dawley rats of both sexes (180–220 g) were obtained from the Experimental Animal Center of the Fourth Military Medical University, Xi'an, China.

### 2.3. Chromatographic Conditions

Chromatographic separation was performed using a Symmetry C_18_ column (50 × 2.1 mm, 5 *μ*m pore size; Waters, Beverly, MA, USA) controlled by a Waters 2695 HPLC system. The isocratic mobile phase was 70 : 30 (v/v) mix of acetonitrile and 0.1% (v/v) aqueous formic acid. The flow rate was 0.2 mL/min. The column temperature was 25°C. The injection volume was 10 *μ*L.

Tandem mass spectroscopy was performed using a Waters Quattro Premier triple-stage/quadrupole mass spectrometer equipped with an ESI interface in the negative ion mode ([M-H]^−^) with multiple reaction monitoring. The source temperature was 110°C. The electrospray capillary voltage was 3.0 kV. Argon served as the collision-dissociation gas (0.18 L/min flow rate), and nitrogen was the desolvation gas (500 mL/min flow rate) [15]. All data were acquired using Masslynx Analyst Software, version 4.1 (Waters).

### 2.4. Preparation of Calibration Curves and Quality-Control (QC) Samples

Stock solutions of DOX and resveratrol were prepared in absolute methanol at concentrations of 100 and 10 *μ*g/mL, respectively. Working solutions were freshly prepared by serial dilution of the stock DOX solution with methanol. The plasma calibration-curve samples were prepared by spiking untreated rat plasma samples (0.2 mL) with 25 ng resveratrol/mL (final concentration) and 0.1 mL of a DOX working solution to yield solutions containing 0.5, 1, 2.5, 10, 50, 200, or 500 ng DOX/mL. The tissue calibration-curve samples were prepared by spiking untreated tissue homogenates (100 mg) with 50 ng/g resveratrol (final concentration) and 0.1 mL of a DOX working solution to yield solutions containing 1, 2.5, 5, 10, 50, 100, or 250 ng DOX/g.

Three QC samples [18] were prepared by spiking untreated rat plasma or tissue homogenates with a DOX working solution so that the final concentrations were 2, 40, or 400 ng/mL for the plasma samples and 5, 25, or 200 ng/g for the tissue samples. To avoid DOX degradation, the stock solutions used for calibration and QC were frozen and stored at −20°C until use.

### 2.5. Sample Preparation

Plasma samples (0.2 mL) from rats that had been dosed with free DOX or a DOX conjugate were mixed with resveratrol (25 ng/mL, final concentration). Tissues from the rats were rinsed with saline and homogenized at 0°C, and then 100 mg of each homogenate was spiked with resveratrol (50 ng/g, final concentration). All homogenates were extracted twice with 2 mL ethyl acetate by vigorously vortexing for 1 min and then centrifuged at 3000 rpm for 5 min at room temperature. The organic layers were removed and evaporated to dryness at 30°C under nitrogen. Each dry residue was dissolved in 100 *μ*L methanol, and then 10 *μ*L of each sample was subjected to LC-MS/MS [19].

### 2.6. Validation of the Method

The linearity of the plasma and tissue calibration curves was considered satisfactory if the associated *R* ^2^ values were >0.99 inclusive of the DOX calibration concentrations.

The recoveries of extracted DOX from the experimental plasma and tissue samples were evaluated by first calculating the ratios of the DOX to resveratrol parent peak areas for the QC samples (*n* = 6) and the experimental samples and then dividing the averaged value for the experimental samples by the averaged QC value.

The intraday uncertainty as a measure of precision was determined using six QC samples at each concentration. The interday uncertainty as a measure of precision was performed over 3 days using six QC samples each day. Uncertainty is expressed as the percentage standard deviation. Six QC samples at each of the three DOX concentrations were used to determine the relative error of the measurements (%) after calculating the observed concentration divided by the nominal concentration. An error within ±15% of the nominal concentration and an uncertainty with a standard deviation of ±15% were considered acceptable.

### 2.7. Rat Pharmacokinetic Study

The rats were fasted overnight with free access to water before the experiment. A dose of DOX or one of the conjugates (DOX equivalent, 5 mg/kg) was intravenously administrated. The rats were sacrificed using diethyl ether 0.08, 0.17, 0.5, 1, 2, 4, 8, 12, or 24 h later. Blood samples were collected from the abdominal aorta and then centrifuged at 3000 rpm to isolate the plasma. Heart, liver, spleen, lungs, and kidneys were removed at 0.5, 2, 8, or 24 h. All samples were stored at −70°C until analysis.

After injection of a DOX conjugate, DOX might exist in its free or conjugated form; therefore, total DOX (free and conjugated DOX) in plasma and tissues was quantified after acid hydrolysis of plasma and tissues as follows [20]. Plasma (100 *μ*L) or tissue (100 mg) was incubated with 50 *μ*L 5 M HCl at 50°C for 2 h, after which 50 *μ*L of resveratrol (20 *μ*g/mL), 50 *μ*L 0.5 M sodium phosphate (pH 7.4), and 50 *μ*L of 1 M NaOH were added at room temperature. The extraction procedure was same as that for the sample preparation described above. Samples were analyzed using an HPLC system with a 2996-photodiode-array detector (Waters, Milford, MA, USA) set at 290 nm after elution from the Waters Symmetry C_18_ column (250 × 4.6 mm, 5 *μ*m pore size). The mobile phase was 0.01 M KH_2_PO_4_/methanol/acetic acid (30 : 70 : 0.3, v/v/v). The flow rate was 1 mL/min. The injection volume was 20 *μ*L.

### 2.8. Statistical Analyses

Each result is expressed as the mean ± SD (*n* = 6). Statistical analysis was performed as a one-way analysis of variance, and comparisons among groups were performed using an independent sample *t*-test. Pharmacokinetic parameters of the DOX conjugates were processed by WinNonlin (version 2.1).

## 3. Results

### 3.1. MS/MS Protocol

Because DOX signals are stronger in the negative ESI mode than in the positive mode, the former was incorporated into our protocol (see Figure S1 in Supplementary Material available online at http://dx.doi.org/10.1155/2013/926584). The parent [M-H]^−^ DOX and resveratrol ions have *m*/*z* values of 542 and 227, respectively, and were selected for MS/MS. The most intense fragmented DOX ion was found at *m*/*z* = 395, whereas that for resveratrol had an *m*/*z* of 143. The monitored range for signal acquisition of the fragmented DOX ions was, therefore, from 542 to 395  *m*/*z*, and that for resveratrol was from 227 to 143  *m*/*z*.

### 3.2. Method Validation

The protocol specificity was assessed using the spiked QC plasma and tissue samples. The retention times for DOX and resveratrol were 2.77 and 2.40 min, respectively. No endogenous peaks that overlapped the DOX and resveratrol peaks were observed (Figure S2).

The ratio of the DOX to resveratrol peak areas (*y*-axis) versus the nominal DOX concentrations (*x-*axis) was plotted and found to have a good linear relationship over the tissue range of 1 to 250 ng/g and the plasma range of 0.5 to 500 ng/mL (*R* ^2^ > 0.99 for all samples). The lower limit of quantification for the DOX plasma and tissue samples was, therefore, set to 0.5 ng/mL and 1 ng/g, respectively. The regression equation for the plasma samples was *y* = 0.1728*x* + 0.0540 (*R* ^2^ = 0.9953) and that for the liver samples was *y* = 0.1932*x* + 0.0210 (*R* ^2^ = 0.9975). Because the slopes and the intercepts of the tissue calibration curves did not vary significantly and the recoveries of DOX from the QC samples also did not differ significantly, we used the calibration curves obtained from the spiked QC liver samples to quantify DOX in all tissues [11].

Measurements of intraday and interday uncertainty, extraction recovery, and error used the QC samples (Tables S1, S2). The intraday and interday uncertainty varied by 13.4%, and the errors ranged from 93.1% to 115.6%.

The extraction method used for sample pretreatment [13] reduces matrix interference, removes impurities, precipitates proteins, and minimizes ion-suppression effects simultaneously, which makes it a suitable means to subsequently measure free DOX in plasma and tissue samples. The extraction recovery for the QC samples ranged from 82.5% to 92.1% for the plasma samples and 81.5% to 95.2% for the tissue samples, showing the robust efficiency of the method.

### 3.3. Blood Distribution

**Figure 1:**
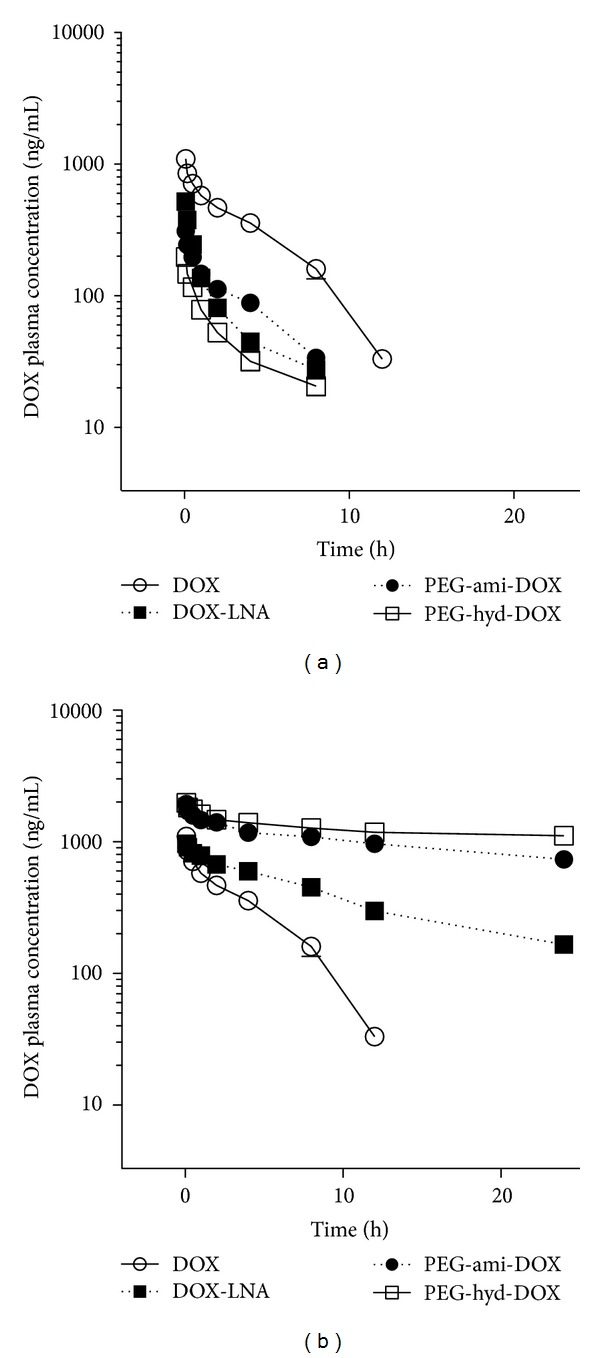
DOX plasma concentration after intravenous administration. (a) Free DOX and (b) total DOX were released from DOX-LNA, PEG-ami-DOX, and PEG-hyd-DOX following a single intravenous administration of 5 mg/kg (DOX equivalent) to SD rats. Each point and bar represents the mean ± SD (*n* = 6).

Because certain DOX side effects are tissue-specific, we surveyed the tissue distribution, including that of blood and that of free and conjugated DOX. After administering DOX (5 mg/kg) or the equivalent amount of DOX as a conjugate by tail-vein injection, the blood distribution was determined (Table 1, Figure 1). Free DOX had a relatively short half-life for elimination (*t*_1/2_ *β*) of 3.13 h and a small AUC_0→∞_ value of 6.27 *μ*g/mL/h, which was a consequence of its low bioavailability and necessitates a larger or more frequent clinical dose to obtain a better therapy index, even though such dosing carries with it a greater risk for toxicity manifested as severe side effects [21].

Smaller amounts of free DOX derived from the conjugates were found in the plasma, meaning that the conjugates were stable in the circulatory system. Among the conjugates, DOX-LNA released more DOX than did either PEG conjugate, with PEG-hyd-DOX releasing the least amount of DOX. The C_max⁡_ value for DOX released from DOX-LNA, PEG-ami-DOX, and PEG-hyd-DOX administration was 521.7, 311.3, and 197.4 ng/mL, respectively, whereas it was 1100.3 ng/mL for unconjugated DOX. Although *t*_1/2_ *β* for DOX released from the conjugates was similar to that of free DOX, the corresponding AUC_0→∞_, CL, and *V*_*c*_ values were significantly smaller, meaning that the type of conjugate determined the free DOX concentration in the blood and its pharmacokinetics (Table 1). Moreover, the concentration of the free and bound DOX in the blood for the conjugates had markedly different pharmacokinetics, with more stable concentration per unit time curves, and an obvious delayed blood clearance (Figure 1). The *t*_1/2_ *β* for total DOX from DOX-LNA, PEG-ami-DOX, and PEG-hyd-DOX was 5.79, 10.22, and 15.18 h, respectively. Therefore, because DOX was bound to the conjugates differently, the pharmacokinetics differed significantly. In addition, the total-DOX AUC_0→∞_ values for DOX-LNA, PEG-ami-DOX, and PEG-hyd-DOX were 21.25, 101.18, and 357.57 *μ*g/mL/h, respectively (*P* < 0.01, compared with the AUC_0→∞_ value for free DOX and *P* < 0.05 for the comparison of the DOX-LNA and the PEG conjugates values.) Although a significant difference in the AUC_0→∞_ values of DOX from PEG-ami-DOX and PEG-hyd-DOX was found (*P* < 0.05), the DOX-PEG conjugates existed for longer periods in the blood than did DOX and DOX-LNA, during which time the introduction of the pH-sensitive bond in PEG-hyd-DOX markedly decreased the release of DOX in the blood. Furthermore, the CL values for total plasma DOX from DOX-LNA, PEG-ami-DOX, and PEG-hyd-DOX were significantly smaller than that of unconjugated DOX (211.58, 45.76, and 12.88 mL/h/kg, resp., versus 721.44 mL/h/kg for DOX), which supports the longer retention and slower plasma elimination rate found for conjugated DOX. Among the conjugates, the two PEG conjugates had a considerably slower release profile as shown by their *t*_1/2_ *β*, AUC_0→∞_, *V*_*c*_, and CL values, which might be attributed to the inertness of PEG leading to a decreased rate of uptake by mononuclear phagocytes and absorption by plasma proteins [21, 22].

### 3.4. Tissue Distribution of Unconjugated DOX and the DOX Conjugates

**Figure 2:**
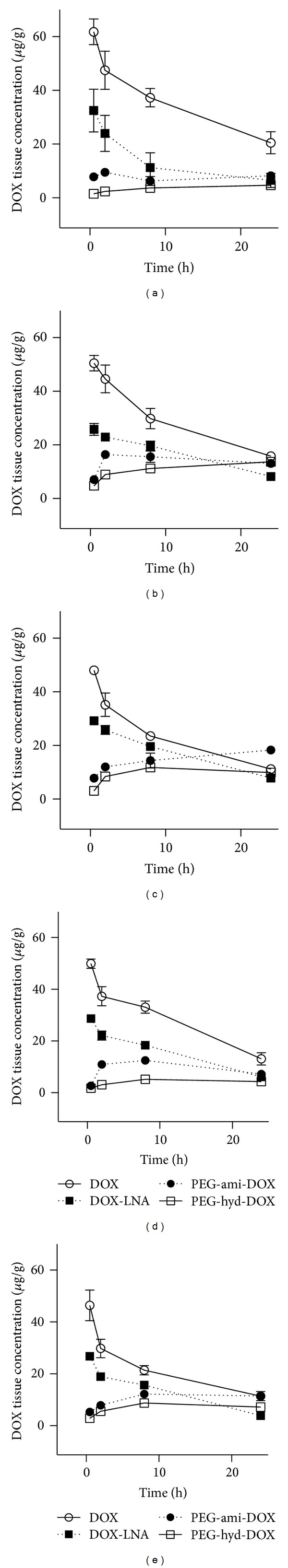
Tissue distribution characteristic of free DOX. Free DOX distribution in (a) heart, (b) liver, (c) spleen, (d) lung, and (e) kidney after intravenous administration of doxorubicin or its conjugates at a dose of 5 mg DOX-equiv./kg. Each point and bar represents the mean ± SD (*n* = 6).

The biodistribution of free DOX from the conjugates was investigated by HPLC/MS-MS with free DOX being used as the control (Figure 2). Because DOX is a small molecule without a targeting moiety, it was more widely distributed in organs than was free DOX from the conjugates.

Similar to the blood results, free DOX and its conjugates were found in the tissues, although the two PEG conjugates had different distributions than did the lower-molecular-weight ones, that is, DOX-LNA and unconjugated DOX. For free DOX, the order of its overall tissue concentration was PEG-hyd-DOX < PEG-ami-DOX < DOX-LNA < DOX (0.5–8 h), and the free DOX concentrations from the conjugates were obviously less in all organs than that found when unconjugated DOX had been injected (*P* < 0.05 at 0.5, 2, and 8 h, *n* = 6). The PEG conjugates released even less DOX than did DOX-LNA (*P* < 0.05 at 0.5 and 2 h). Because DOX damages the heart, we measured the amounts of free DOX released by the conjugates in the hearts. The C_max⁡_ value of free DOX released by DOX-LNA, PEG-ami-DOX, and PEG-hyd-DOX was 32.5, 9.5, and 4.7 *μ*g/g, respectively, whereas when free DOX had been injected, the value was 61.8 *μ*g/g. A small accumulation of free DOX in the heart should reduce the risk of heart injury; DOX from the two PEG conjugates may be less toxic than that from DOX-LNA because it appears that DOX existed mostly as conjugated to PEG in the heart. For DOX from DOX-LNA, its distribution in normal tissues was not significantly different from that of conjugated DOX, which indicates that this small molecular conjugate only decreased the cumulative amount of free DOX without changing the distribution profile. The amounts of free DOX released by the PEG conjugates were greater in the liver and spleen than in the other tissues, which may be a consequence of the polymeric PEG structure [23, 24]. The PEG conjugates also had different time-concentration curves, as their free DOX *t*_max⁡_ values were greater than those for unconjugated DOX or DOX-LNA. Among the conjugates, PEG-hyd-DOX performed best in terms of reducing the free DOX accumulation in the organs, especially the heart (Figure 2(a)), which indicated that it might induce the lowest level of side effects as a consequence of its pH-response profile and high molecular weight.

**Figure 3:**
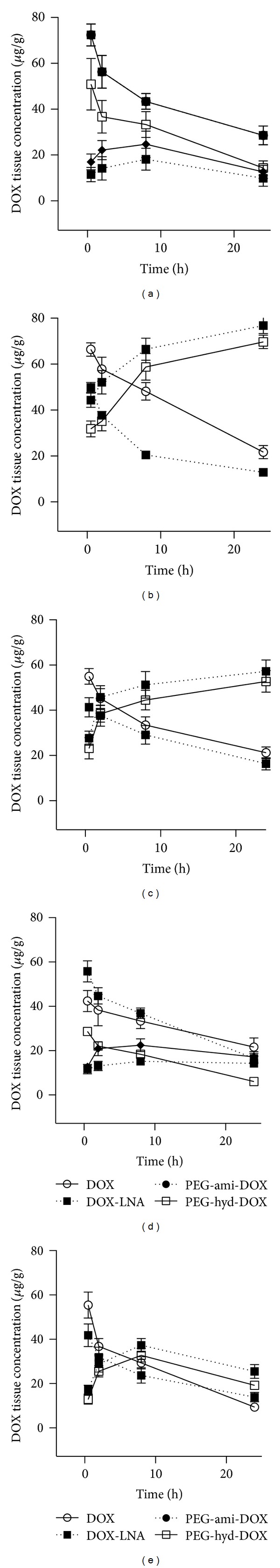
Tissue distribution characteristic of total DOX. Total DOX distribution in (a) heart, (b) liver, (c) spleen, (d) lung, and (e) kidney after intravenous administration of doxorubicin conjugates at a dose of 5 mg DOX-equiv./kg. Each point and bar represents the mean ± SD (*n* = 6).

We also determined the total DOX concentration to further clarify the distribution of the conjugates in tissues (Figure 3). As with the free DOX distributions, total DOX from DOX-LNA was the same within the uncertainty in all tissues, which again suggested that this small-molecule carrier did not change the distribution profile. Total DOX from the PEG conjugates accumulated to a greater extent in the spleen and liver, with the concentrations of total DOX being liver > spleen > kidney > heart > lung, whereas the order for unconjugated DOX was heart > lung > spleen > kidney > liver. Total DOX released from the conjugates distribution in the heart also decreased more obviously than the distribution of DOX control group, with the PEG conjugates showing much lower accumulation in the heart compared with DOX-LNA (*P* < 0.05 at 0.5, 2, and 8 h). These results further supported the hypothesis that the PEG conjugates would constitute the less toxic DOX delivery systems.

## 4. Discussion

To counter the low selectivity and efficacy of DOX, we have synthesized three new DOX conjugates with antitumor and tumor-targeting activities. In this report, we focused on the relationship between the pharmacokinetics and chemistry of these conjugates. The conjugates are covalently bound to DOX and release DOX *in vivo* after administration. We found significant differences in their pharmacokinetics that possibly can be attributed to their chemistries. The two PEG conjugates released DOX more slowly and had different tissue distribution profiles compared with unconjugated DOX, possibly owing to their polymeric structures. However, all conjugates decreased the accumulation of free DOX in the circulatory system and in solid organs, which suggests that they may have a lower toxicity than unmodified DOX. PEG-hyd-DOX was the most stable and released little free drug in the circulatory system owing to its pH-sensitive structure. Our results suggest a direct relationship between the structures of the conjugates and their performances *in vivo*.

Many analytic methods have been reported for the determination of DOX concentration. For this report, we developed and validated a sensitive, rapid, and reproducible LC-MS/MS method. The high sensitivity and specificity of this method guarantee that DOX can be quantified in blood and tissue samples, which should be useful when assessing the utility of other possible DOX-delivery systems, for example, nanoparticles, micelles, and liposomes.

We previously showed that DOX-LNA and PEG-hyd-DOX have better antitumor activity than did unconjugated DOX *in vitro* and *in vivo*; although administration of PEG-ami-DOX increased the intracellular DOX concentration, its *in vitro* antitumor efficacy was much poorer owing to an incomplete release of free DOX after cellular uptake and organellar release. These results suggest that the chemical structure of the DOX carrier is crucial for efficacy and are supported by the results presented herein. DOX-LNA, a low-molecular-weight DOX conjugate containing DOX conjugated to *α*-linoleic acid, a polyunsaturated fatty acid, had greater uptake efficiency by tumor cells and increased antitumor efficacy [15, 16]. When compared with its clearance from the circulatory system and its organ distribution, DOX-LNA exhibited much greater accumulation of free DOX and had a shorter *t*_1/2_ *β* than did the PEG conjugates. Therefore an increase in side effects may result from the accumulation of free DOX released from DOX-LNA and its low bioavailability associated with LNA. Because DOX and LNA are linked via an amide bond, hydrolysis of that bond might occur in the blood, which would increase the amount of free DOX released before encountering the tumor. Moreover, DOX-LNA is a low-molecular-weight compound and has a pharmacokinetic profile similar to that of unconjugated DOX. Therefore, although LNA may target DOX appropriately because it does not stably maintain DOX in the complex, it is a failure as a delivery system [25]. We chose PEG 6000 as the polymeric carrier for DOX delivery because of its well-known biocompatibility, passive tumor-targeting profile, and inertness in the circulatory system [26]. The two PEG conjugates released DOX relatively slowly, escaped recognition by the reticuloendothelial system, had comparable stabilities before encountering tissue cells, and should be less toxic than DOX-LNA on account of their decreased distributions in normal tissues. Of the two PEG conjugates, PEG-hyd-DOX had a much better *in vivo* retention time, a better AUC_0→∞_ value, and other pharmacokinetic parameters, with a lower free DOX organ distribution. These characteristics of PEG-hyd-DOX can be attributed to the acid-liable bond between DOX and PEG. Under the blood physiological condition of pH 7.4, PEG-hyd-DOX should be stable and be hydrolyzed only in the moderately acidic environment (pH 5.0) found in lysosomes and endosomes of tumor cells where DOX would be rapidly released in large concentrations [17, 27, 28]. The pH-sensitive characteristic of PEG-hyd-DOX is probably responsible for its stability in blood, its low distribution in healthy organs, and its statistically significant antitumor activity, suggesting that PEG-hyd-DOX should be highly efficacious and not toxic when administered clinically. Conversely, release of DOX from PEG-ami-DOX is not pH dependent, and more DOX may be released inappropriately from this carrier.

The structural integrity of the delivery system in the circulation and in healthy tissues is critical for antitumor activity. Ideally, three elements are required for a delivery system: a specific tumor-targeting profile, a stable chemical or physical structure, and release of the drug only in tumor cells. PEG-hyd-DOX possesses all three elements.

We evaluated three tumor-targeting DOX-conjugates that had been prepared using different chemical strategies with respect to pharmacokinetics and organ distribution of DOX release *in vivo*. In comparison with DOX administered in free form, these conjugates released a relatively smaller amount of DOX in healthy tissues, were not rapidly removed from the circulatory system, and had greater bioavailabilities, suggesting that these conjugates would have lower toxicity than does DOX. PEG-hyd-DOX exhibited the best performance owing to the pH-sensitive covalent bond between PEG and DOX. It is apparent that the chemical structure of a carrier is crucial for the design of a chemotherapeutic agent with its stability prior to arriving at its target being the most important aspect for improved therapeutic efficacy and safety.

## Supplementary Material

The mass spectrum and MRM chromatograms of doxorubicin were illustrated in the supplementary material. The mass spectrum of doxorubicin was showed in Figure s1, the monitored range for signal acquisition of the fragmented DOX ions was from 542 to 395 m/z. MRM chromatograms of doxorubicin and *I.S.* (resveratrol) in rat plasma and tissue samples were showed in Figure s2. The retention times for doxorubicin and resveratrol were 2.77 min and 2.40 min, respectively. In addition, the method of LC-MS/MS were also evaluated by precision, accuracy, and extraction recoveries of doxorubicin.

## Figures and Tables

**Table 1:** Pharmacokinetic parameters of doxorubicin and its conjugates.

Parameters	DOX	DOX-LNA		PEG-ami-DOX		PEG-hyd-DOX	
		Free DOX	Total DOX	Free DOX	Total DOX	Free DOX	Total DOX
*t*_1/2_(*β*) (h)	3.13 ± 1.11	3.22 ± 0.91	5.79 ± 1.69*	3.53 ± 1.21	10.22 ± 2.39**	3.73 ± 1.34	15.18 ± 2.81**
AUC_0→*∞*_ (*μ*g/mL/h)	6.27 ± 2.33	4.12 ± 1.12	21.25 ± 9.77*	3.82 ± 0.38	101.18 ± 21.33**	1.91 ± 0.22	357.57 ± 44.29^∗∗,#^
CL (mL/h/Kg)	721.44 ± 95.08	577.21 ± 29.18	211.58 ± 32.18*	468.29 ± 38.04	45.76 ± 18.44**	411.17 ± 47.02	12.88 ± 7.23**
*V*_*C*_ (mL/kg)	1368.21 ± 77.43	979.33 ± 66.97	168.77 ± 27.44**	903.97 ± 74.62	78.52 ± 19.55**	788.73 ± 59.38	22.29 ± 8.48^∗∗,#^

*P < 0.05; **P < 0.01, as compared with DOX formulation*;* ^#^ *P* < 0.05, as compared with PEG-ami-DOX. (*n* = 6).
