# Supplementary material for: Deposition of Doxorubicin in Rats following Administration of Three Newly Synthesized Doxorubicin Conjugates
Source: Biomed Res Int. 2013 Dec 5;2013:926584. doi: 10.1155/2013/926584 (PMC3870082; doi:10.1155/2013/926584)
Supplement: Supplementary file 1 — The mass spectrum and MRM chromatograms of doxorubicin were illustrated in the supplementary material. The mass spectrum of doxorubicin was showed in Figure s1, the monitored range for signal acquisition of the fragmented DOX ions was from 542 to 395 m/z. MRM chromatograms of doxorubicin and I.S. (resveratrol) in rat plasma and tissue samples were showed in Figure s2. The retention times for doxorubicin and resveratrol were 2.77 min and 2.40 min, respectively. In addition, the method of LC-MS/MS were also evaluated by precision, accuracy, and extraction recoveries of doxorubicin. [file 926584.f1.pdf]

Supplementary data

Fig s1. Mass spectrum of doxorubicin. The monitored range for signal acquisition of the fragmented DOX ions was from 542 to 395 m/z.

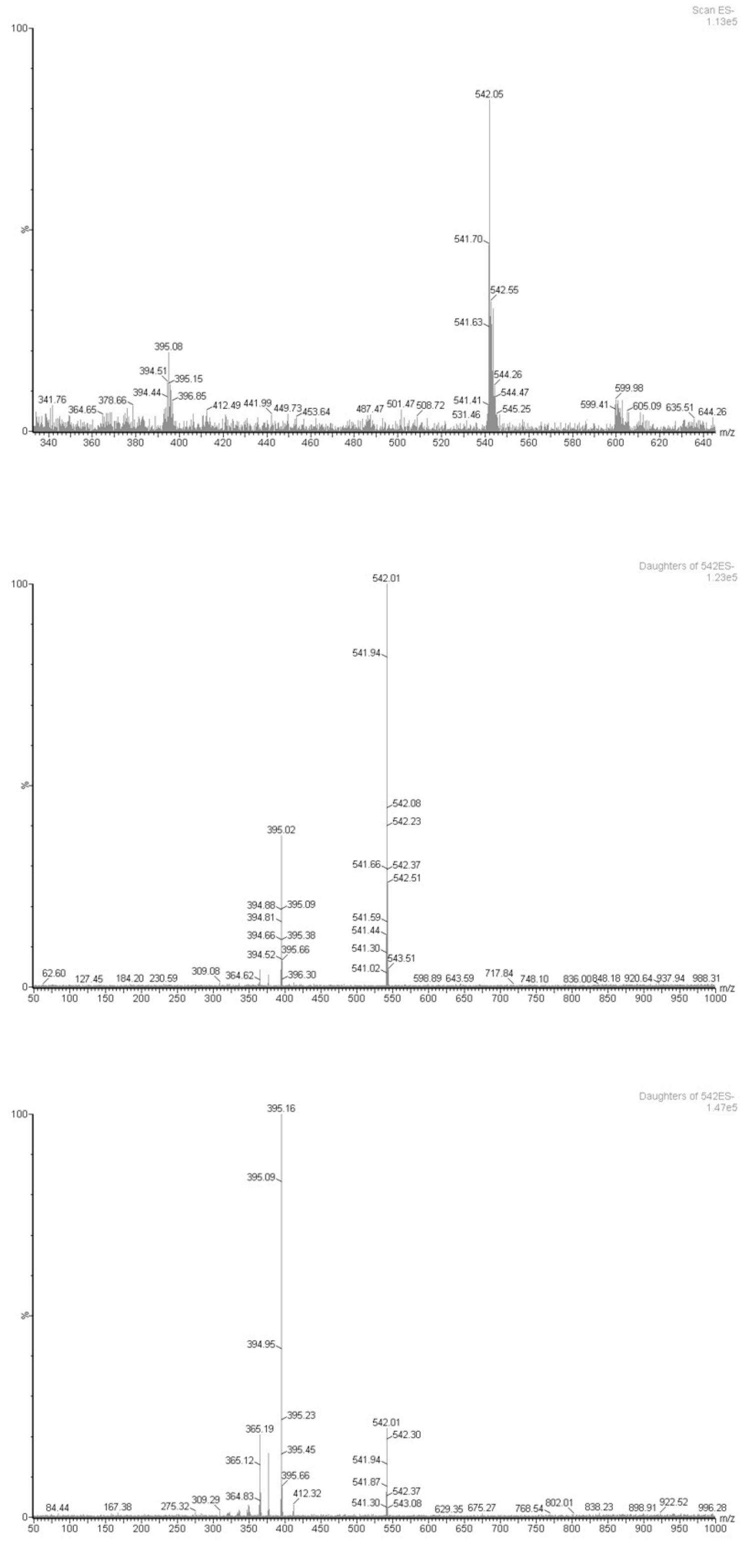

Fig s2. MRM chromatograms of doxorubicin and I.S. (resveratrol) in rat plasma and tissue samples. The retention times for doxorubicin and resveratrol were 2.77 min and 2.40 min, respectively.

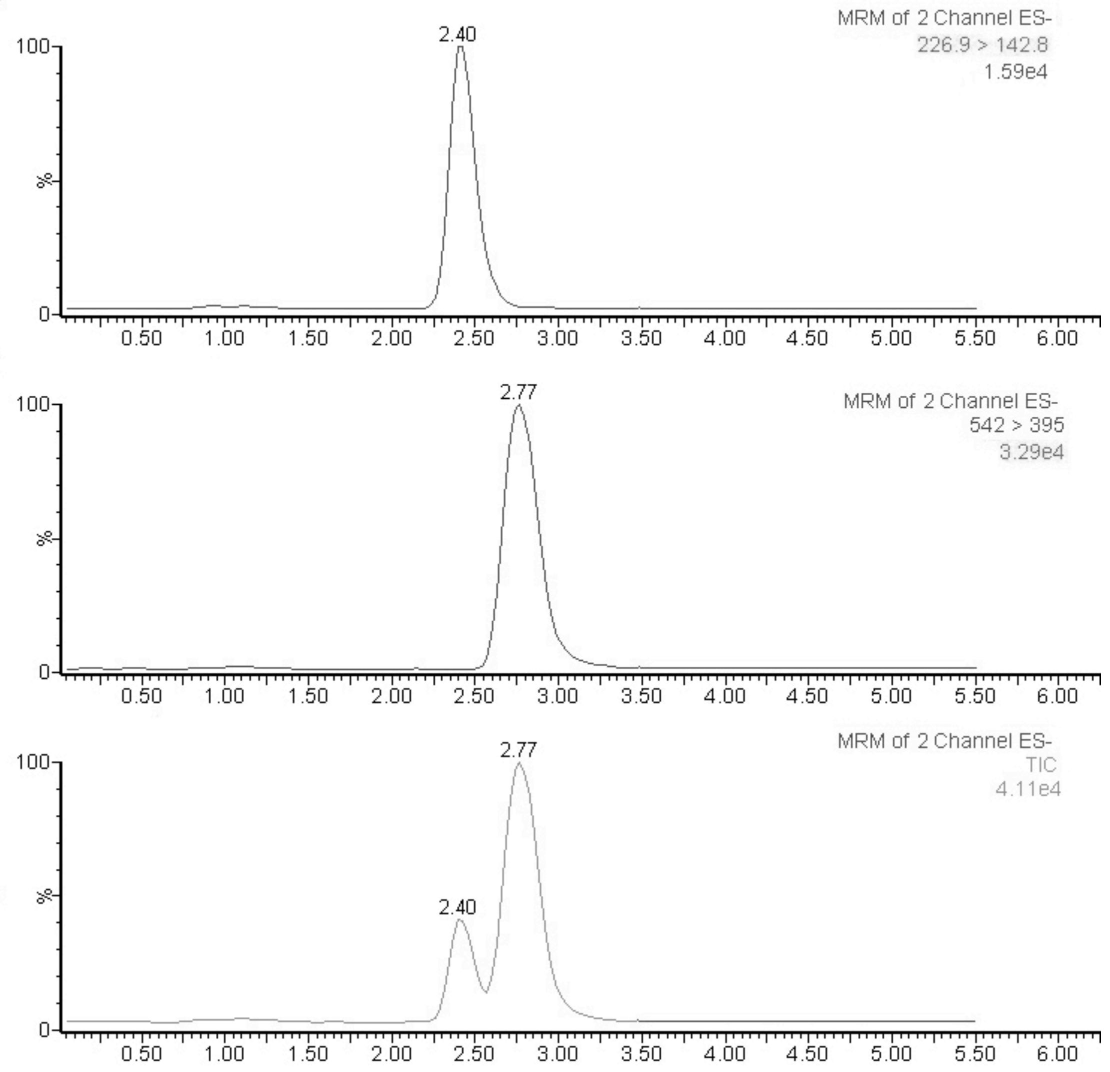

Table s1. Precision and accuracy of doxorubicin using LC-MS/MS method.

Spiked samples	Spiked concentration	Intra-day		Inter-day	
		Accuracy%	Precision (RSD%)	Accuracy%	Precision (RSD%)
Plasma (ng/ml)	2	115.6	12.5	107.7	13.4
	40	99.6	8.9	97.8	9.6
	400	100.3	4.7	98.2	6.4
Heart (ng/g)	5	100.8	11.5	100.1	12.6
	25	93.1	7.5	97.2	9.3
	200	95.9	3.9	96.8	7.2
Liver (ng/g)	5	101.3	8.9	98.3	10.3
	25	100.7	7.5	95.2	9.9
	200	93.6	5.2	94.7	6.1
Spleen (ng/g)	5	97.8	5.2	96.2	7.8
	25	103.5	3.7	97.1	4.4
	200	96.7	2.9	95.4	3.6
Lung (ng/g)	5	98.6	10.3	95.1	11.8
	25	99.2	7.5	97.6	8.3
	200	97.3	5.7	96.5	6.1
Kidney (ng/g)	5	105.2	9.4	99.4	8.1
	25	93.7	3.6	95.7	5.5
	200	95.2	2.3	96.8	3.9

Table s2. Extraction recoveries of doxorubicin using LC-MS/MS method.

Spiked amount	Plasma (%)	Heart (%)	Liver (%)	Spleen (%)	Lung (%)	Kidney (%)
Low dose	83.6±2.8	93.2±4.4	91.2±2.7	82.9±5.1	95.2±3.3	90.5±3.1
Middle dose	82.5±7.1	85.4±1.7	85.7±3.4	94.7±6.1	90.5±2.6	85.3±2.1
High dose	92.1±5.2	87.1±4.7	81.5±3.6	82.7±2.3	88.4±3.5	90.4±3.1

Low, middle and high dose for plasma and other tissues was 2, 40, 400ng/ml or 5, 25, 200ng/g respectively.
